# Genomic Insertion of a Heterologous Acetyltransferase Generates a New Lipopolysaccharide Antigenic Structure in *Brucella abortus* and *Brucella melitensis*

**DOI:** 10.3389/fmicb.2018.01092

**Published:** 2018-05-25

**Authors:** Estrella Martínez-Gómez, Jonas Ståhle, Yolanda Gil-Ramírez, Amaia Zúñiga-Ripa, Mona Zaccheus, Ignacio Moriyón, Maite Iriarte, Göran Widmalm, Raquel Conde-Álvarez

**Affiliations:** ^1^Instituto de Salud Tropical, Instituto de Investigación Sanitaria de Navarra, Departamento de Microbiología y Parasitología, Universidad de Navarra, Pamplona, Spain; ^2^Department of Organic Chemistry, Arrhenius Laboratory, Stockholm University, Stockholm, Sweden

**Keywords:** lipopolysaccharide (LPS), bacterial pathogenesis, bacteria, vaccine development, virulence factor, antigen, brucellosis, *Brucella*

## Abstract

Brucellosis is a bacterial zoonosis of worldwide distribution caused by bacteria of the genus *Brucella*. In *Brucella abortus* and *Brucella melitensis*, the major species infecting domestic ruminants, the smooth lipopolysaccharide (S-LPS) is a virulence factor. This S-LPS carries a *N*-formyl-perosamine homopolymer *O*-polysaccharide that is the major antigen in serodiagnostic tests and is required for virulence. We report that the *Brucella* O-PS can be structurally and antigenically modified using *wbdR*, the acetyl-transferase gene involved in *N*-acetyl-perosamine synthesis in *Escherichia coli* O157:H7. *Brucella* constructs carrying plasmidic *wbdR* expressed a modified O-polysaccharide but were unstable, a problem circumvented by inserting *wbdR* into a neutral site of chromosome II. As compared to wild-type bacteria, both kinds of *wbdR* constructs expressed shorter O-polysaccharides and NMR analyses showed that they contained both *N*-formyl and *N*-acetyl-perosamine. Moreover, deletion of the *Brucella* formyltransferase gene *wbkC* in *wbdR* constructs generated bacteria producing only *N*-acetyl-perosamine homopolymers, proving that *wbdR* can replace for *wbkC*. Absorption experiments with immune sera revealed that the *wbdR* constructs triggered antibodies to new immunogenic epitope(s) and the use of monoclonal antibodies proved that *B. abortus* and *B. melitensis wbdR* constructs respectively lacked the A or M epitopes, and the absence of the C epitope in both backgrounds. The *wbdR* constructs showed resistance to polycations similar to that of the wild-type strains but displayed increased sensitivity to normal serum similar to that of a *per* R mutant. In mice, the *wbdR* constructs produced chronic infections and triggered antibody responses that can be differentiated from those evoked by the wild-type strain in S-LPS ELISAs. These results open the possibilities of developing brucellosis vaccines that are both antigenically tagged and lack the diagnostic epitopes of virulent field strains, thereby solving the diagnostic interference created by current vaccines against *Brucella*.

## Introduction

The Gram-negative bacteria of the genus *Brucella* are the etiological agents of brucellosis, a zoonosis that causes abortions and infertility in domestic livestock and wildlife and a grave and debilitating disease in humans. Eradicated in a handful of countries, this disease is endemic or increasing in many areas of Asia, Africa, and America due to changing breeding conditions and agricultural intensification, thus representing a serious problem in developing economies throughout the world (Jones et al., 2013; McDermott et al., 2013; Ducrotoy et al., 2015; Lai et al., 2017).

The brucellae are facultative intracellular parasites that, in addition to the ability to control the intracellular trafficking and to adapt their metabolism to the nutrients available in the replicative niche (Martirosyan et al., 2011), owe their pathogenicity to structural peculiarities of the outer membrane (OM) that reduce detection by innate immunity (Lapaque et al., 2005; Barquero-Calvo et al., 2007). The OM of *B. abortus, B. suis*, and *B. melitensis* (*Brucella* smooth [S] species found in ruminants and swine), carry a S-LPS poorly recognized by cell receptors, complement and bactericidal peptides. The *Brucella* lipid A is a diaminoglucose disaccharide substituted with very long acyl chains that is linked to a core oligosaccharide carrying a characteristic glucosamine lateral branch (Conde-Álvarez et al., 2012; Kubler-Kielb and Vinogradov, 2013b; Fontana et al., 2016). These lipid A and core structures differ from those of typical LPS, reduce and mask the PAMP of LPS and are thus critical for virulence (Lapaque et al., 2005, 2006; Conde-Álvarez et al., 2012). In *Brucella* S-LPS, mannose and *N*-acetyl-quinovosamine link the core to the O-PS, a homopolymer of *N*-formyl-perosamine (4-formamido-4,6-dideoxy D-mannose) in various proportions of α-(1→2)- and α-(1→3)-linkages usually but not always with a terminal cap of at least a tetra-saccharide unit containing α-(1→3)-linked *N*-formyl-perosamine (Perry and Bundle, 1990; Kubler-Kielb and Vinogradov, 2013a; Zaccheus et al., 2013; Fontana et al., 2016). In at least *B. abortus, B. suis*, and *B. melitensis*, the O-PS is involved in the resistance to killing by non-immune serum, reduces the access of complement and bactericidal peptides to OM targets and its loss causes attenuation (Lapaque et al., 2005; González et al., 2008). Although these O-PS properties could be accounted for by topological effects of O-PS length such as a steric hindrance of soluble elements of innate immunity and of non-specific adhesion to host cells (Lapaque et al., 2005; González et al., 2008), it is also possible that *N*-formyl-perosamine is specifically needed for virulence. Testing this hypothesis would require a specific manipulation of the chemical structure of the O-PS in live bacteria. Moreover, such a manipulation could also modify the epitopic structure of the O-PS. Taking into account the prominent role of this section of the S-LPS in serological diagnosis (Ducrotoy et al., 2016), the development of an antigenic tag for S brucellosis vaccines affecting the diagnostic epitopes of virulent field strains could open the possibility of solving the diagnostic interference created by current brucellosis vaccines, a major problem in the eradication of brucellosis in ruminants (Ducrotoy et al., 2018).

Although to the best of our knowledge genetic modification of *Brucella* O-PS has never been attempted, the genetics of *Brucella* O-PS has been largely elucidated (González et al., 2008; Fontana et al., 2016), and this opens the possibility of manipulating its structure. Since the genes required for perosamine synthesis (*manA*, *manB*, *gmd* and *per*) and its formylation (*wbkC*) are indispensable for O-PS expression, gene replacement seems the simplest strategy, and potential tools are genes carried by the few Gram-negative bacteria endowed of heteropolymeric O-PS with *N*-acylated perosamine (Perry and Bundle, 1990). Here we report that *wbdR*, an O-PS acetyltransferase gene of *Escherichia coli* O157:H7, can replace *wbkC* and generates *Brucella* constructs carrying *N*-acetyl-perosamine. We also describe the effect that this O-PS modification has on the epitopic structure of *B. abortus* and *B. melitensis* and discuss the practical implications in the development of new brucellosis vaccines.

## Materials and Methods

### Bacterial Strains and Growth Conditions

The bacterial strains and plasmids used are listed in Supplementary Table S1. Bacteria were routinely grown in standard tryptic soy broth (TSB; Biomérieux, Solna, Sweden) or agar (TSA; Pronadisa, Laboratorios Conda, Spain) either plain or supplemented with Km at 50 μg/ml, or Cm at 20 μg/ml, or nalidixic acid (Nal) at 25 μg/ml. When needed, media were supplemented with 5% sucrose. All strains were stored in skimmed milk (Scharlau, Barcelona, Spain) at -80°C.

### DNA Manipulations

Plasmid and chromosomal DNA were extracted with Qiaprep spin Miniprep (Qiagen GmbH, Hilden, Germany) and Ultraclean Microbial DNA Isolation Kit (Mo Bio Laboratories), respectively. When needed, DNA was purified from agarose gels using a Qiack Gel extraction kit (Qiagen). DNA sequencing was performed by “Servicio de Secuenciación del Centro de Investigación Médica Aplicada” (Pamplona, Spain) and “Unidad de Genómica del Instituto de Parasitología y Biomedicina López-Neyra” (Granada, Spain). Primers (Supplementary Table S2) were synthesized by Sigma-Genosys Ltd. (Haverhill, United Kingdom).

### *wbdR*-Constructs

Primers *wbdR* attB Fw (5′ggggacaagtttgtacaaaaaagcaggcttcATGA ATTTGTATGGTATTTTTGGT3′) and *wbdR* attB Rv (5′ggggaccactttgtacaagaaagctgggtcTTAAATAGATGTTGGCGA TCTT3′) were designed based on the sequence of *E. coli* O157:H7^1^ and according to Gateway Cloning Technology^®^ (Invitrogen, Barcelona, Spain) instructions. Both primers were used to amplify *wbdR* from the start to the stop codon using *E. coli* O157:H7 DNA as template, and the resulting product was cloned into pDONR221 (Invitrogen) by site-specific recombination, generating pYRI-5. Then, *wbdR* from pYRI-5 was transferred by site-specific recombination to pRH001 (Hallez et al., 2007). The new plasmid (pYRI-6) was introduced in Ba-parental (*B. abortus* 2308W) by conjugation (Conde-Álvarez et al., 2006) to obtain Ba-p*wbdR* (Supplementary Table S1).

To obtain a stable *B*. *abortus*-*wbdR* construct, gene *wbdR* with the 300 bp upstream region containing its promoter was amplified from *E. coli* O157:H7 using primers *wbdR* Fw: 5′TTCCCCGGGGGAgaagttcgccacagtaaatcgaa3′ and *wbdR* Rv: 5′TTCCCCGGGGGAttaaatagatgttggcgatctt3′, and cloned in pGEM-T Easy^®^ (Promega, Madison, WI, United States) to obtain pYRI-21. The construction was verified by sequencing. Then, the *Eco*RI fragment of pYRI-21 containing *wbdR* and its promoter was subcloned into the corresponding site of pUC18R6KT-miniTn7TKm (Llobet et al., 2009) to obtain pYRI-27 (pUC18R6KT-miniTn7T-Km-P*wbdR*). The miniTn7 vector carrying *wbdR* with its own promoter was inserted into Ba-parental chromosome II by the method of Choi et al. (2005) and Choi and Schweizer (2006) modified as follows. First pYRI-27 was introduced in *E. coli* S17.1 λpir and then transferred to *Brucella* using an *E. coli* S17.1 λpir (pYRI-27)-*E. coli* HB101 (pRK2013)-*E. coli* SM10 λpir (pTNS2)-Ba-parental four-parental mating. The resulting Ba::Tn7*wbdR*Km^R^ construct was examined by PCR for the correct insertion and orientation of miniTn7 between genes *glmS* and *recG* using the following primers: (i) GlmS_B (5′-GTCCTTATGGGAACGGACGT-3′) and Ptn7-R (5′-CACAGCATAACTGGACTGATT-3′) for insertion downstream *glmS*; (ii) Ptn7-L (5′-ATTAGCTTACGACGCTACACCC-3′) and RecG (5′-TATATTCTGGCGAGCGATCC-3′) for insertion downstream *recG*; and (iii) GlmS_B and RecG that only amplify the intergenic region in the absence of the mini-Tn7. The presence of only one copy of the miniTn7 was determined by Southern-blot and sequencing.

To obtain a *wbdR* construct with no Km resistance (Ba::Tn7*wbdR*), a non-polar *kmR* mutant of Ba::Tn7*wbdR*Km^R^ was constructed by overlapping PCR using the Km cassette of pUC18R6KT-miniTn7TKm as template. Primers *kmR*-F1 (5′-AGGAAGCGGAACACGTAGAA-3′) and *kmR*-R2 (5′-AATCATGCGAAACGATCCTC-3′) amplified a 318-bp fragment including 312-bp upstream of the *kmR* start codon and codons 1 to 2 of the *kmR* ORF, and primers *kmR*-F3 (5′-gaggatcgtttcgcatgattTTCTTCTGAGCGGGACTCTG-3′) and *kmR*-R4 (5′-TGGTCCATATGAATATCCTCCTTA-3′) amplified a 268-bp fragment including codons 262–264 of the *kmR* ORF and 256 bp downstream of the *kmR* stop codon. Both fragments were ligated by overlapping PCR using oligonucleotides *kmR*-F1 and *kmR*-R4 for amplification, and the complementary regions between *kmR*-R2 and *kmR*-F3 for overlapping. The resulting fragment, containing the *kmR* deletion allele, was cloned into pCR2.1 (Invitrogen) and subcloned into the *Eco*RI site of the suicide plasmid pNPTS138-Cm (Addgene, LGC Standards, Teddington, United Kingdom) to generate plasmid pRCI-65. This suicide plasmid was used to delete the *kmR* gene of Ba::Tn7*wbdR*Km^R^ using the allelic exchange by double recombination (Conde-Álvarez et al., 2006). Deletion of *kmR* was checked with oligonucleotides *kmR*-F1 and *kmR*-R4.

A Ba::Tn7*wbdR*Δ*wbkC* mutant potentially expressing only the *wbdR* encoded acetyltransferase was constructed by PCR overlap using genomic DNA of Ba-parental as template. Primers *wbkC*-F1 (5′-AGGTGGCGACAAACGAATAA-3′) and *wbkC*-R2 (5′-GCCCATGCCAATCAAGGT-3′) amplified a 393-bp fragment including codons 1–29 of the *wbkC* ORF (BAB1_0540), as well as 306 bp upstream of the *wbkC* start codon, and primers *wbkC*-F3 (5′-accttgattggcatgggcAGATGGTCGGAAGTCCAGATT-3′) and *wbkC* -R4 (5′-TCTGAACTCGGCTGGATGAC-3′) amplified a 434-bp fragment including codons 212–259 of the *wbkC* ORF and 287-bp downstream of the *wbkC* stop codon. Both fragments were ligated by overlapping PCR using oligonucleotides *wbkC*-F1 and *wbkC*-R4 for amplification, and the complementary regions between *wbkC*-R2 and *wbkC*-F3 for overlapping. The fragment containing the *wbkC* deletion allele was cloned into pCR2.1 and subcloned into the *Bam*HI and the *Xba*I sites of the suicide plasmid pJQK (Scupham and Triplett, 1997). The resulting mutator plasmid pYRI-31 was used to delete the *wbkC* gene of Ba::Tn7*wbdR* by allelic exchange (Conde-Álvarez et al., 2006). The resulting colonies were screened by PCR with primers *wbkC*-F1 and *wbkC*-R4, which amplify a fragment of 827 bp in the mutant and a fragment of 1373 bp in the parental strain.

Bme::Tn7*wbdR*Km^R^ was obtained using the modified miniTn7 site-specific integration vector technology (see above). To obtain Bme::Tn7*wbdR* and Bme::Tn7*wbdR*Δ*wbkC* the suicide plasmids pRCI-65 and pYRI-31 (see above and Supplementary Table S1) were used.

### Stability of *wbdR* in Ba-*pwbdR* and Ba::Tn7*wbdR*

The constructs Ba-p*wbdR* and Ba::Tn7*wbdR* were grown in the presence of Km and Cm (plasmid antibiotic markers) or Km (transposon antibiotic marker), adjusted to an O.D._600_ = 0.109 and CFU counted on TSA and TSA Km/Cm or TSA and TSA Km (transfer 0). A 100 μl aliquot of the culture was inoculated into 10 ml of TSB without antibiotics, the broth incubated for 48 h and CFU counted on TSA and TSA Km/Cm or on TSA and TSA Km. The process was serially repeated five times.

### Bacteriological Characterization, Antibiotic Sensitivity and Growth Curves

Colonial morphology, urease, and sensitivity to R/C phage were determined following established *Brucella* typing procedures (Alton et al., 1988). S/R colony morphology was studied by the crystal violet dye exclusion test and acriflavine agglutination (Alton et al., 1988). Autoagglutination was evaluated by measuring the O.D._600_ of bacterial suspensions in TSB after 6 and 14 days of static incubation at 37°C. To obtain inocula for growth curves, bacteria were first grown in 10 ml of TSB supplemented with antibiotics in a 50 ml flask, incubated with orbital stirring at 37°C for 18 h, harvested by centrifugation and resuspended at a O.D._600_ of 0.1 in TSB. Then, aliquots were dispensed into Bioscreen plates (200 μl/well) which were incubated in a Bioscreen C (Lab Systems Quesada, Capital Federal, Argentina) with continuous shaking at 37°C. Growth was monitored at 420–580 nm every 30 min over a 65 h-period (control wells contained sterile TSB). All experiments were performed in triplicate.

### LPS and PS Preparations

Crude S-LPS from Ba-parental or *B. abortus wbdR* constructs was obtained by the method of Leong et al. (1970) with modifications. For the wild-type strain, the phenol phase of a phenol-water extract was precipitated with 2 volumes of methanol to obtain the total crude S-LPS. For *wbdR* constructs, this classical precipitation step failed to yield the total of the S-LPS and precipitation was achieved with 6 volumes of methanol. Alternatively, the S-LPS remaining in the supernatant of the methanol precipitation was dialyzed and freeze-dried. These crude S-LPS were resuspended (10 mg/ml) in 175 mM NaCl, 0.05% NaN_3_, 0.1M Tris-HCl (pH 7.0), digested once with nucleases (50 μg/ml each of DNase-II type V, and RNase [Sigma, St. Louis, MO, United States] 30 min at 37°C) and then three times with proteinase K (50 μg/ml, 3 h at 55°C). Finally, the S-LPS was recovered by ultracentrifugation (6 h at 100,000 × *g* in a Beckman Ti70 rotor) (Aragón et al., 1996).

Purified LPS were used to obtain the PS by mild acid hydrolysis in 1% SDS acetate buffer 10 mM (pH 4.5) at 100°C for 2 h, a method that splits the lipid A-core linkage. After cooling at room temperature and brought to pH 7 with 0.2 M NaOH, lipid A and debris were pelleted (10 min at 5000 × *g*) and the supernatant was dialyzed against distilled water, lyophilized and chromatographed in acetate-pyridine buffer on Sephadex G50. Fractions were analyzed for PS by in gel immunoprecipitation in 10% NaCl, 100 mM borate (pH 8.3) (Diaz-Aparicio et al., 1993).

### SDS-PAGE and NMR Spectroscopy

LPS were analyzed in 15% polyacrylamide gels (37.5:1 acrylamide/methylenebisacrylamide ratio) in Tris-HCl-glycine and stained by the periodate-alkaline silver method (Tsai and Frasch, 1982).

The ^1^H NMR spectrum of the Ba-parental PS (5 mg in 0.55 mL D_2_O) was recorded at 25°C and the 1D and 2D NMR spectra of the Ba-p*wbdR* (10 mg in 0.5 mL D_2_O), Ba::Tn7*wbdR* (5 mg in 0.55 mL D_2_O) and Ba::Tn7*wbdR*Δ*wbkC* PS (10 mg in 0.55 mL D_2_O) were recorded at 47°C, 25°C and 25°C, respectively. All experiments were performed on Bruker AVANCE 500 MHz or Bruker AVANCE III 700 MHz spectrometers; both equipped with 5 mm TCI Z-Gradient CryoProbes. ^1^H chemical shifts were referenced to internal sodium 3-trimethylsilyl-(2,2,3,3-^2^H_4_)-propanoate (TSP, δ_H_ 0.00) and ^13^C chemical shifts were referenced to external dioxane in D_2_O (δ_C_ 67.40). Data processing was performed using vendor-supplied software. The assignments of the ^1^H and ^13^C resonances of the Ba-parental, Ba-p*wbdR*, Ba::Tn7*wbdR* and Ba::Tn7*wbdR*Δ*wbkC* PS were obtained by analysis of ^1^H and ^13^C NMR spectra together with a multiplicity-edited^1^H,^13^C-HSQC experiment (Parella et al., 1997), ^1^H,^1^H-TOCSY using mixing times ranging from 10 to 120 ms (Bax and Davis, 1985), as well as a band-selective constant time ^1^H,^13^C-HMBC experiment of Ba-p*wbdR* PS, which was used to correlate the *N*-acyl groups (Claridge and Pérez-Victoria, 2003).

### Antibodies and Immunological Tests

To obtain a serum reacting with O-PS carrying both *N*-formyl- and *N*-acetyl-perosamine (i.e., the Formyl-Acetyl serum), rabbits were inoculated intravenously with 1 mg/ml of phenol-inactivated Ba-p*wbdR* cells. Then, similar doses were administrated intraperitoneally 2 and 4 days later and the animals bled 3 weeks later. The animals (2.5 Kg New-Zealand female rabbits; San Bernardo, Spain) were kept in cages with water and food *ad libitum* in the animal facilities of “Centro de Investigación en Farmacobiología aplicada” (University of Navarra). Rabbits were handled, bled and euthanized according to the Spanish and European recommendations (RD 1201/2005; directive 86/609/ECC), and the protocols approved by the Animal Health Care Department of University of Navarra.

To obtain a serum reacting only with *N*-acetyl-carrying O-PS (i.e., the Acetyl serum) or *N*-formyl-carrying O-PS (i.e., the Formyl serum), the Formyl-Acetyl serum was absorbed with Ba-parental or Ba::Tn7*wbdR*Δ*wbkC* cells, respectively. Absorption was performed at 4°C with continuous stirring overnight. Cells were removed (13,200 rpm, 10 min, Eppendorf 5415R centrifuge), and the absorption repeated with incubation for 4 h at room temperature. As a control of absorption, Formyl-Acetyl serum was absorbed with Ba-p*wbdR* cells following the same procedure. Finally aliquots of Formyl-Acetyl or Acetyl serum were subsequently absorbed with cells from rough mutant BaΔ*per* to obtain Formyl-Acetyl-OPS or Acetyl-OPS, respectively.

Monoclonal antibodies 42D2 (C/Y-A>M; preferentially reacting with *B. abortus* and *Yersinia enterocolitica* O:9 O-PS), and 33H8 (C/Y-A = M; equally reacting with *B. abortus*, *B. melitensis* and *Y. enterocolitica* O:9 O-PS) were provided by INGENASA (Madrid, Spain) and the reactivities verified by ELISA using LPS of *B. abortus* 2308, *B. melitensis* 16M and *Yersinia enterocolitica* O:9. Monoclonal antibody A15-6B3 (or 6B3) M; reacting with *B. melitensis* but not *B. abortus* O-PS (Laurent et al., 2004) was a generous gift of J. J. Letesson (University of Namur).

For coagglutination, staphylococci were prepared and sensitized with the appropriate serum (Kronvall, 1973), and the bacteria in 4-6 colonies resuspended in 25 μl of saline on a glass slide and mixed with an equal amount of the sensitized staphylococci.

For Western Blots, SDS-PAGE gels (see above) were electrotransferred onto nitrocellulose sheets (Whatmann, Dassel, Germany), blocked with 3% PBS with 0.05% Tween 20 (PBS-T) overnight, and washed with PBS-T. Immune sera or monoclonal antibodies (see below) were diluted in this same buffer and, after incubation overnight at room temperature and washing the membranes with PBS-T, bound immunoglobulins were detected with peroxidase-conjugated goat anti-rabbit immunoglobulin (Nordic Immunological Laboratories, Tilburg, Netherlands) or peroxidase labeled protein G for mouse sera and 4-chlorine-1-napthol-H_2_O_2_.

For ELISA, 96-well ELISA plates (Thermo Scientific, Waltham, MA, United States) were coated with 2.5 μg/ml LPS resuspended in PBS overnight at 4°C. Plates were then washed extensively with PBS-T, and incubated with serial dilutions of the appropriate sera or with monoclonal antibodies at 37°C for 5 h. Then, the plates were washed extensively with PBS-T and bound antibodies were detected with peroxidase-labeled protein G (Alonso-Urmeneta et al., 1988, 1998). The peroxidase activity was detected with 2,2′-azino-bis(3-ethylbenzthiazoline-6-sulphonic acid) (ABTS)/H_2_O_2_. After 15 min, the color was measured in a microtiter plate reader (Multiescanex, Thermo Scientific, Waltham, MA, United States) at 405 nm.

### Infections in Mice

Female BALB/c mice (Harlan Laboratories; Bicester, United Kingdom) were kept in cages with water and food *ad libitum* under P3 biosafety conditions in the facilities of “Centro de Investigación Médica Aplicada” (registration code ES31 2010000132) 2 weeks before and during the experiments. The procedures were in accordance with the current European (directive 86/609/EEC) and Spanish (RD 53/2013) legislations, supervised by the Animal Welfare Committee of the University of Navarra, and authorized by the “Gobierno de Navarra” (CEEA 045/12). To prepare inocula, TSA or TSA-Km grown bacteria were harvested, adjusted spectrophotometrically (O.D._600_ = 0.170) in 10 mM in PBS and diluted in the same buffer to approximately 5 × 10^5^ CFU/ml (exact doses were assessed retrospectively). For each bacterial strain, five mice were intraperitoneally inoculated with 0.1 mL/mouse. Eight weeks later, mice were bled and CFU numbers in spleen were determined. Statistical significance was evaluated using one-way *ANOVA* followed by *Dunnett’s* test.

### Sensitivity to the Bactericidal Action of Normal Serum

Exponentially growing bacteria were adjusted to 10^4^ CFU/mL in saline and dispensed in triplicate in microtiter plates (45 μL per well) containing fresh normal bovine or ovine serum (90 μL/well). After 15 and 90 min for the bovine serum or 15 and 45 min for the ovine serum at 37°C, brain heart infusion broth was dispensed (200 μL/well), mixed with the bacterial suspension and 100 μL plated on TSA.

### Sensitivity to Polycationic Bactericidal Peptides

Bacterial sensitivity was measured as the effect of increasing concentrations of polymyxin B and poly-l-ornithine on cell viability as described elsewhere (Martínez de Tejada et al., 1995).

### Surface Charge

The surface charge density was measured as the electrophoretically effective potential (Zeta potential) as previously described (González et al., 2008). Bacteria were grown in TSB, inactivated with 0.5% phenol, washed and resuspended in 1 mM CsCl, 10 mM HEPES 10 mM (pH 7.2) at an O.D._600_ of 0.2. Measurements were performed at 25°C in a Zetamaster instrument using the PCS 1.27 software (Malvern Instruments Ltd., Malvern, United Kingdom).

## Results

### Identification of *wbdR*: A Perosamine Acetyltransferase for Cloning in *Brucella*

The literature describes at least twelve Gram-negative bacteria with O-PS that contain substituted perosamine (Supplementary Table S3). Among them, *E. coli* O157:H7 was selected because its O-PS contains *N*-acetyl-perosamine (D-Rha*p*4NAc) [O-PS repeating unit: →2)-α-D-Rha*p*4NAc-(1→3)-α-L-Fuc*p*-(1→4)-β-D-Glc*p*-(1→3)-α-D-Gal*p*NAc-(1→] and, moreover, the complete genomic sequence is available. Indeed, synthesis of *N*-acetyl-perosamine requires an acetyl-transferase and a putative one, encoded by gene *wbdR*, was found in the O-PS gene cluster of *E. coli* O157:H7. Apparently, no equivalent gene is found in the O-PS cluster of other bacteria carrying *N*-acetyl-perosamine (Albermann and Beuttler, 2008).

### Insertion of *wbdR* Into *B. abortus* Chromosome Is Required to Generate a Stable *wbdR* Construct (Ba::Tn7*wbdR*)

*wbdR* was amplified and cloned into pRH001 to obtain plasmid pYRI-6, which was then introduced into wild-type *B. abortus* 2308W (Ba-parental) to obtain strain Ba-p*wbdR* (Supplementary Table S1). Since plasmids can be lost in the absence of selective pressure and pYRI-6 carries Km/Cm resistance, the stability of Ba-p*wbdR* was studied by serial passage in broth without antibiotics, and aliquots plated on TSA and TSA Km/Cm. One log reduction in CFU/ml was observed on the TSA Km/Cm plates with respect to the TSA plates (Supplementary Figure S1), showing that the construct was not fully stable. To circumvent this problem, a technology that uses the miniTn7 site-specific integration vector was adapted to *Brucella* to integrate *wbdR* into chromosome II (See Experimental Procedures). The new construct (Ba::Tn7*wbdR*Km^R^; Supplementary Table S1) was stable in the absence of antibiotic selective pressure (Supplementary Figure S1).

Ba::Tn7*wbdR*Km^R^ contained *wbkC*, the parental formyltransferase gene, so that it could retain at least part of *N*-formyl-perosamine in its O-PS or no acetyl-perosamine if WbkC activity displaced that of WbdR. To obtain a strain lacking WbkC, the *kmR* cassette was first removed to obtain Ba::Tn7*wbdR* and then *wbkC* deleted to generate Ba::Tn7*wbdR*Δ*wbkC.*

### *B. abortus wbdR* Constructs Produce S-LPS Carrying *N*-acetyl-perosamine

**FIGURE 1:**
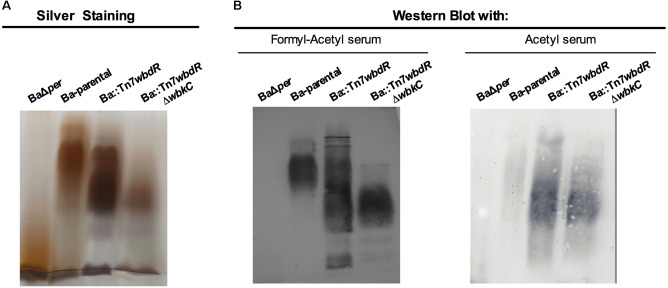
*Brucella abortus wbdR* constructs express an S-LPS that displays a new antigenic structure. SDS-PAGE followed by silver staining **(A)** and Western blots of SDS-proteinase K extracts of the indicated bacteria **(B)**. Ba*Δper* is a mutant defective in perosamine synthesis that expresses only R-LPS.

Ba-p*wbdR*, Ba::Tn7*wbdR* and Ba::Tn7*wbdR*Δ*wbkC* were identical to the parental strain in colony morphology and growth (Supplementary Figure S2), oxidase and urease tests and dye sensitivity. Moreover, they did not agglutinate with acriflavine or autoagglutinated (Supplementary Figure S3), and they excluded crystal violet and were resistant to phage R/C (specific for the O-PS-lacking rough [R] brucellae), a set of properties characteristic of strains expressing S-LPS (Alton et al., 1988). Since *wbkC* (i.e., formyl-transferase gene) mutants lack O-PS (Godfroid et al., 2000), these results strongly suggest that Ba::Tn7*wbdR*Δ*wbkC* carries a surface O-PS and, therefore, that *wbdR* can replace for *wbkC*, a hypothesis studied by SDS-PAGE analysis of LPS obtained by the SDS-proteinase K protocol. The LPS of Ba::Tn7*wbdR* and Ba::Tn7*wbdR*Δ*wbkC* (**Figure 1A**) and Ba::Tn7*wbdR*Km^R^ and Ba-p*wbdR* (not shown) contained the typical R-LPS and S-LPS fractions present in the parental strain. However, SDS-PAGE also showed that the average molecular weight of the S-LPS fraction of the *wbdR* constructs was lower than that of Ba-parental (**Figure 1A**).

**FIGURE 2:**
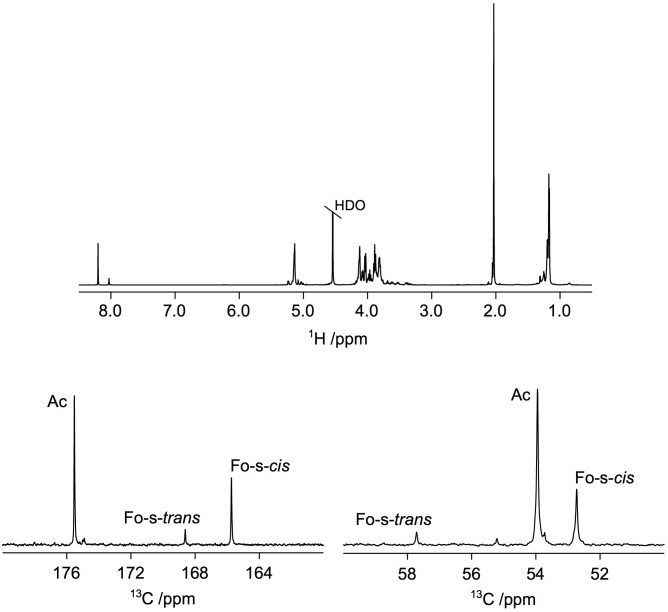
Introduction of *wbdR* into *B. abortus* leads to the expression of *N*-acetyl-perosamine in the O-PS. ^1^H NMR spectrum **(Top)** and selected ^13^C NMR spectral regions corresponding to carbonyl region **(Lower Left)** and region for C4 resonances **(Lower Right)** of *N*-formylated and *N*-acylated perosamine in strain Ba-p*wbdR.*

The ^1^H NMR spectrum of the Ba-parental PS (i.e., the core-O-PS obtained by hydrolysis of LPS) (Supplementary Figure S4) was fully consistent with that reported before for *B. abortus* 2.13 (Supplementary Figure S2 in González et al., 2008) showing, among others, resonances from *N*-formyl groups but absence of resonances for *N*-acetyl groups, except for a very small one at *δ*_H_ 2.07 consistent with the presence of *N*-acetyl-quinovosamine (the primer for O-PS polymerization) previously investigated in detail using *B. abortus* 2.13. Also, the ^13^C NMR spectrum of Ba-parental PS (Supplementary Figure S4) and ^13^C chemical shifts (Supplementary Table S4) were consistent with those of a *B. abortus* 1119-3 PS (Perry and Bundle, 1990). In contrast, in addition to the expected resonances from *N*-formyl groups at *δ*_H_ 8.03 and 8.20 (∼0.3 equivalents), the ^1^H NMR spectrum of the Ba-p*wbdR* PS revealed a conspicuous resonance from an *N*-acetyl group at *δ*_H_ 2.03 (∼0.6 equivalents) (**Figure 2**). Moreover, the ^13^C NMR spectrum showed resonances in the carbonyl region at *δ*_C_ 175.5 from the *N*-acetyl group and at *δ*_C_ 168.6 and 165.7 from the two conformations of the *N*-formyl group, (Engström et al., 2017) in the region for nitrogen-carrying carbon atoms at *δ*_C_ 53.9 from C4 of perosamine carrying the *N*-acetyl group and at *δ*_C_ 57.7 and 52.7 from C4 of perosamine substituted by the *N*-formyl group (**Figure 2**) as well as a resonance from the methyl group of the *N*-acetyl group at *δ*_C_ 23.0 supporting the proposed substitution pattern. This was confirmed by a ^1^H,^13^C-BS-CT-HMBC NMR experiment in which heteronuclear correlations over two and/or three bonds could be observed; in particular, correlations were observed at *δ*_C_/*δ*_H_ 165.7/3.96 and 168.6/3.40 between the carbonyl atom of the *N*-formyl group and H4 of perosamine as well as at *δ*_C_/*δ*_H_ 175.5/3.89 and 175.5/2.03 between the carbonyl atom of the *N*-acetyl group and H4 of perosamine and the methyl protons of the *N*-acetyl group, respectively, fully consistent with NMR chemical shifts of perosamine with these substituents (Kenne et al., 1988).

**FIGURE 3:**
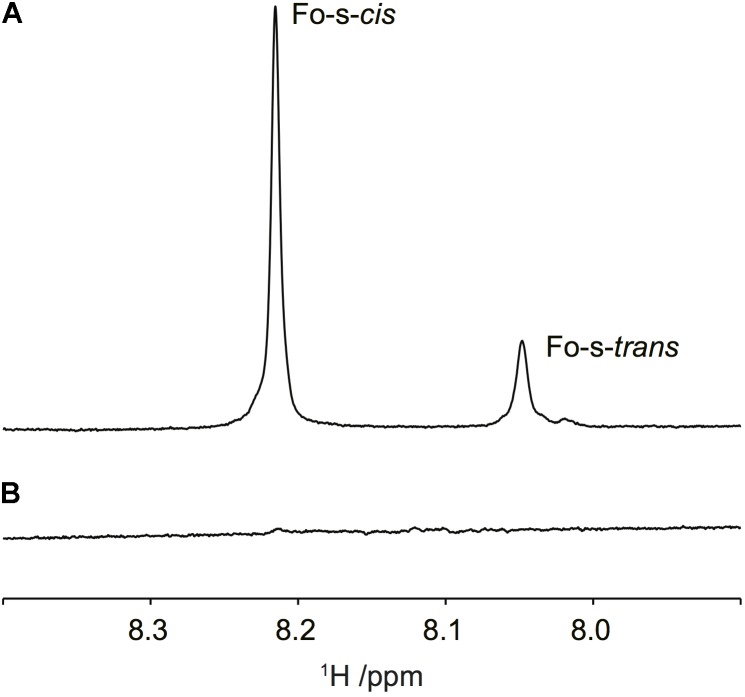
Ba::Tn7*wbdR*Δ*wbkC* PS lacks *N*-formyl groups in the O-PS. ^1^H NMR spectral region of formyl resonances of **(A)** Ba::Tn7*wbdR* PS and **(B)** Ba::Tn7*wbdR*Δ*wbkC* PS.

Subsequent NMR analysis showed that an *N*-acetyl group was also present in the Ba::Tn7*wbdR* PS since a singlet in the ^1^H NMR spectrum was observed at 2.05 ppm. Formyl group substitution was still evident from resonances at 8.05 and 8.22 ppm (**Figure 3A**). These findings were supported by ^13^C NMR resonances at, *inter alia*, 165.7, 168.5, and 175.6 ppm (cf. the above Ba-p*wbdR* PS). In strain Ba::Tn7*wbdR*Δ*wbkC*, in which the formyl-transferase gene has been deleted, a prominent ^1^H NMR resonance at *δ*_H_ 2.05 confirmed the presence of an *N*-acetyl group in the PS. Notably, and most importantly, any resonances from *N*-formyl groups in the ^1^H spectral region at ∼8 ppm were completely absent (**Figure 3B**) confirming the successful transformation into an *N*-acetyl-only substituted PS, additionally supported by ^13^C NMR data, *inter alia*, a resonance at 175.6 ppm.

Taken together, the above results show that cloning of *E. coli wbdR* in *B. abortus* resulted in expression of S-LPS molecules whose O-PS contain *N*-formyl plus *N*-acetyl-perosamine residues or only *N*-acetyl-perosamine depending on the simultaneous presence of *wbkC* and *wbdR* or of only *wbdR*, respectively. The degree of polymerization of the *N*-acetyl-perosamine containing O-PS was, however, reduced when compared to the parental O-PS.

### The O-PS of *B. abortus wbdR* Constructs Displays a New Antigenic Structure Lacking the *Brucella* C Epitopes

**FIGURE 4:**
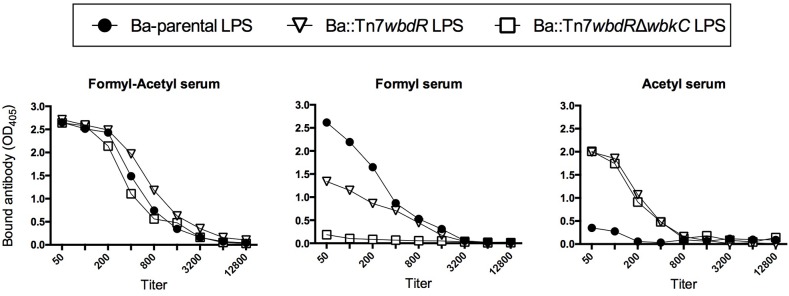
*N*-acetyl-perosamine generates new epitopes in the S-LPS. The ELISAs were performed with the S-LPS of Ba-parental or *B. abortus wbdR*-constructs and anti Ba-*pwbdR* rabbit sera either plain (Formyl-acetyl serum) or adsorbed with Ba::Tn7*wbdR*Δ*wbkC* (Formyl serum) or with Ba-parental (Acetyl serum).

**FIGURE 5:**
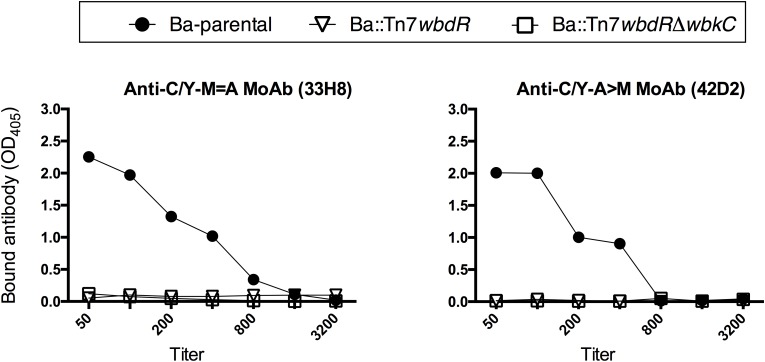
*N*-acetyl-perosamine alters the epitopic structure of S-LPS. Upper panels: Reactivity with monoclonal antibodies of C/Y-A = M and C/Y-A>M specificities in an ELISA performed with the S-LPS of Ba-parental or *B. abortus wbdR*-constructs.

To probe for epitopic changes, polyclonal immune sera specifically recognizing O-PS carrying only *N*-formyl-perosamine, both *N*-formyl-perosamine and *N*-acetyl-perosamine or only *N*-acetyl-perosamine (henceforth anti-Formyl, anti-Formyl-Acetyl, and anti-Acetyl sera, respectively) were obtained by immunization and cross-absorption (see Material and Methods). By coagglutination, only the bacteria containing an intact *wbkC* reacted with the anti-Formyl or anti-Formyl-Acetyl sera. On the other hand, only the *wbdR* constructs agglutinated with the anti-Acetyl serum, and titration of serial dilutions of cell suspensions showed that the reactivity of Ba::Tn7*wbdR*Δ*wbkC* with this serum was slightly higher (1/32 titer) than that of Ba::Tn7*wbdR* or Ba-p*wbdR* (1/16 titer). These analysis proved the surface exposure of the O-PS antigens and, taking into account that polyclonal sera represent a wide spectrum of specificities, avidities and titers, they prove the existence of surface immunogenic epitope(s) associated with the expression of *N*-acetyl-perosamine and the loss of those associated with *N*-formyl-perosamine in Ba::Tn7*wbdR*Δ*wbkC*. To prove that the new epitopes were in fact carried by the S-LPS, we used the purified molecule. iELISA (**Figure 4**) showed epitopic changes, and Western-blots (**Figure 1B**) also demonstrated that the differences in average molecular weight observed by SDS-PAGE (**Figure 1A**) corresponded in fact to O-PS heterogeneity. None of the above results changed when the same sera were absorbed with cells of the R mutant BaΔ*per* (Supplementary Figure S5). Altogether, these results support the conclusion that the epitopes generated by *N*-acetyl-perosamine were absent from wild-type *B. abortus* and that the *N*-acetyl-perosamine O-PS homopolymer of Ba::Tn7*wbdR*Δ*wbkC* lacked the *N*-formyl-perosamine related epitopes present in the parental strain and the Ba::Tn7*wbdR* construct. Although the O-PS of brucellae carries overlapping epitopes that are all detected with polyclonal sera, monoclonal antibodies allow for analyses with more refined structural implications. Thus, we probed the O-PS with monoclonal antibodies of A, M and C epitopes specificities. In contrast to the Ba-parental S-LPS, the S-LPS from *wbdR*-constructs did not react with the C/Y-A = M (Cby33H8) and C/Y-A>M (42D2) monoclonal antibodies (**Figure 5** and Supplementary Figure S6). Ba-parental is a biovar 1 strain that lacks the M epitope (Alton et al., 1988; Douglas and Palmer, 1988) and, as expected, the reactivity of the antibody to the M epitope A156B3 was negative for all these S-LPS (data not shown).

### *B. abortus wbdR* Constructs Display Increased Sensitivity to Normal Serum but Not to Bactericidal Polycations

**FIGURE 6:**
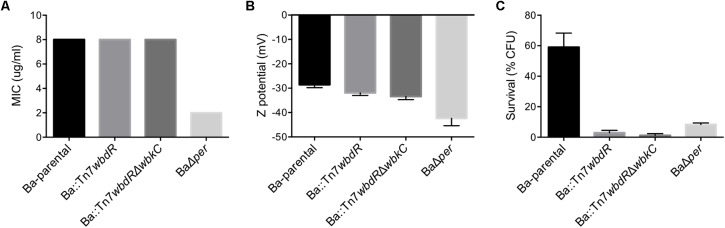
*Brucella abortus wbdR* constructs display increased sensitivity to normal serum but not to bactericidal polycations. Polymyxin B sensitivity (representative results of three independent experiments) **(A)**, surface charge (Z potential) (mean of ± SD of three independent experiments) **(B)** and survival after 90 min of incubation in normal bovine serum (media ± standard error of three technical replicates representative of three independent experiments) **(C)** of Ba-parental, Ba::Tn7*wbdR*, Ba::Tn7*wbdR*Δ*wbkC* and the R mutant BaΔ*per.*

S brucellae but not R mutants are resistance to the bactericidal action to polycations and complement in non-immune serum (Lapaque et al., 2005). The minimal inhibitory concentration of polymyxin B was the same for Ba-parental, Ba::Tn7*wbdR* and Ba::Tn7*wbdR*Δ*wbkC* (**Figure 6A**), and the lack of differences was confirmed with poly-L-ornithine (not shown). Consistent with this phenotype, Ba::Tn7*wbdR* and Ba::Tn7*wbdR*Δ*wbkC* showed only a small increase in negative surface charge as compared to Ba-parental (**Figure 6B**). On the other hand, Ba::Tn7*wbdR* and Ba::Tn7*wbdR*Δ*wbkC* were more sensitive than Ba-parental to the killing action of bovine normal serum and strikingly this susceptibility was similar to that of the R mutant BaΔ*per* (**Figure 6C**). This increased sensitivity was confirmed with sheep normal serum (not shown).

### *B. abortus wbdR* Constructs Produce Chronic Infections in Mice That Trigger Antibodies to the New Epitopes

**FIGURE 7:**
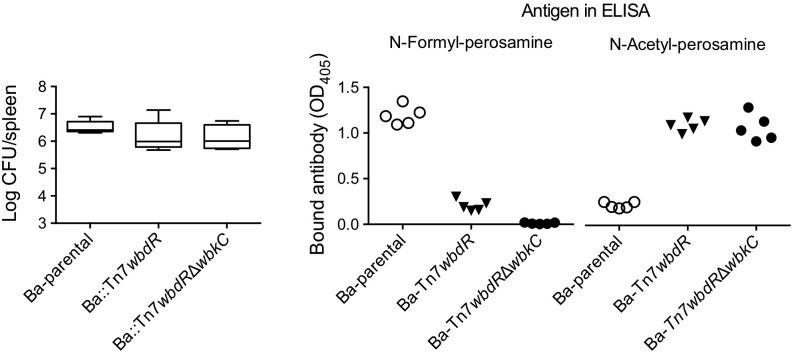
*Brucella abortus wbdR* constructs produce chronic infections in mice that trigger antibodies to the new epitopes. **(Left)** Bacterial loads in the spleens of BALB/c mice at 8 weeks post-infection. **(Center** and **Right)** Reactivity of sera from mice infected with Ba-parental, Ba::Tn7*wbdR* or Ba::Tn7*wbdR*Δ*wbk*C in ELISA performed with the S-LPS of Ba-parental (*N*-Formyl-perosamine iELISA) or Ba::Tn7*wbdR*Δ*wbk*C (N-Acetyl-perosamine iELISA). The ODs correspond to the serum dilution (1:50) showing the maximal discrimination between the wild-type and the *wbdR* constructs.

The above-described experiments were complemented with analyses in the mouse model. As shown in **Figure 7**, the strains carrying *N*-acetyl O-PS kept the ability to multiply in the spleens and persisted in the chronic phase, a characteristic of virulent S brucellae. To test whether the infection triggers antibodies corresponding to the above-described epitopic changes, we tested the sera of these animals. Whereas those infected with Ba-parental developed antibodies strongly reacting in an iELISA with the wild-type *N*-formyl-perosamine LPS, the sera of mice infected with *wbdR* constructs displayed almost no reaction (**Figure 7**), and the reverse picture was obtained in an iELISA with the *N*-Acetyl-perosamine LPS from Ba::Tn7*wbdR*Δ*wbkC* (**Figure 7**).

### The *wbdR*-Encoded Acetyl-transferase Is Also Active in *B. melitensis*

When the Tn7 technology was used to insert *wbdR* in the chromosome II of *B. melitensis* 16M (Bme-parental), the resulting Bme::Tn7*wbdR* construct was also S according to the standard tests for S and R brucellae (Alton et al., 1988). Like its *B. abortus* counterpart, this construct coagglutinated with the anti-Formyl-Acetyl, anti-Acetyl and anti-Formyl sera showing that its O-PS contained *N*-acetyl and *N*-formyl-perosamine. Moreover, when *wbkC* was deleted, the resulting Bme::Tn7*wbdR*Δ*wbkC* strain coagglutinated with the anti-Formyl-Acetyl and anti-Acetyl sera but not with the anti-Formyl serum.

Bme::Tn7*wbdR* and Bme::Tn7*wbdR*Δ*wbkC* synthetized a S-LPS with a S fraction of average molecular weight lower than that of the parental strain (Supplementary Figure S7A), being in this regard also similar to the *B. abortus* equivalents. Also, whereas the S-LPS of Bme-parental, Bme::Tn7*wbdR* and Bme::Tn7*wbdR*Δ*wbkC* reacted with the anti-Formyl-Acetyl serum, only the S-LPS of the *wbdR*-constructs reacted with the anti-Acetyl serum (Supplementary Figure S7B). In addition, Bme::Tn7*wbdR*Δ*wbkC* was the only strain that produced a LPS that did not react with the anti-Formyl serum (Supplementary Figure S7B). With respect to the M and C epitopes carried by *B. melitensis* 16M (a biovar 1 strain) (Alton et al., 1988; Douglas and Palmer, 1988), the monoclonal antibodies Cby33H8 (C/Y epitope) and A156B3 (M epitope) did not react with the LPS of Bme::Tn7*wbdR*Δ*wbkC* (Supplementary Figure S7B). As expected, the reactivity of the monoclonal antibody 42D2 to the A>M epitope was negative with all S-LPS obtained in the *B. melitensis* background (Supplementary Figure S7B). These results show that expression of *wbdR* in a *B. melitensis* background results in the synthesis of O-PS carrying *N*-acetyl-perosamine with epitopic modifications that parallel those observed in the *B. abortus* background.

## Discussion

The results presented in this work demonstrate that *wbdR*, the acetyl-transferase gene involved in the synthesis of the *N*-acetyl-perosamine of the heteropolymeric O-PS repeating unit of *E. coli* O157:H7, can replace for the autochthonous *N*-formyl-transferase gene *wbkC* in representative strains of *B. abortus* and *B. melitensis* biovars 1. In all *Brucella* examined thus far that carry *N*-formyl-perosamine in the O-PS, the sugar linkages vary from almost exclusively α-(1→2) to various proportions of α-(1→2) and α-(1→3), and the arrangement and proportion of these linkages relate to the overlapping A, M, C and C/Y epitopes whose distribution varies among the different *Brucella* serovars (Alton et al., 1988; Meikle et al., 1989; Weynants et al., 1997; Kubler-Kielb and Vinogradov, 2013a; Zaccheus et al., 2013; Zygmunt et al., 2015; Ducrotoy et al., 2016). The O-PS of *B. abortus* 1119-3, a typical biovar 1 strain, contains over 95% α-(1→2)-linkages and is A, C, C/Y, and that of *B. melitensis* 16M, which is M, C, C/Y, displays a 4:1 proportion of α-(1→2) and α-(1→3)-linkages (Bundle et al., 1987; Meikle et al., 1989). Since *wbdR* was functional in both backgrounds it can be proposed that the polymerization of *N*-acetyl-perosamine by the *Brucella* O-PS glycosyltransferases takes place no matter the kind of linkage [α-(1→2) or α-(1→3)] in the native O-PS. Therefore, even though the experiments were carried out in two strains, it seems likely that *wbdR* can replace for *wbkC* in other S-*Brucella* species that carry *N*-formyl-perosamine homopolymers in the S-LPS (i.e., *B. suis*, *B. ceti*, *B. pinnipedialis*, *B. neotomae*, and *B. microti*). However, such an independence of the kind of linkage does not mean that the autochthonous O-PS biosynthetic machinery is as efficient in synthetizing the wild-type and the N-acetyl-perosamine O-PS. We observed that the degree of O-PS polymerization is reduced in the latter, and the present state of knowledge on gram-negative O-PS biosynthesis makes only possible to speculate on the causes. First, it is conceivable that the activities of the heterologous acyl-transferase, which belongs to a taxonomically distant bacterium, and the native enzymes (the glycosyltransferases for formylperosamine polymerization) are not optimal in the heterologous environment and substrates, respectively. Moreover, although to the best of our knowledge systems regulating the length of homopolymeric OPS (*wzm*/*wzt* dependent) are not known for any bacteria, it could be that such systems work differently on heterologous sugars and/or are affected by a reduced flow of precursors. The lack of stability of the Ba-p*wbdR* may also be explained by these hypothetical biosynthetic and export defects.

**FIGURE 8:**
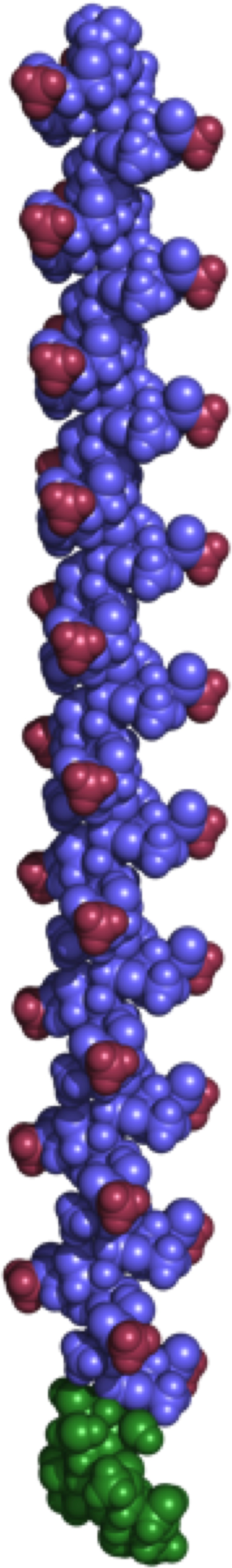
Novel epitopes are accessible for antibodies in the O-PS of Ba::*Tn7wbdRΔwbkC*. The polysaccharide structure containing 30 residues of →2)-α-D-Rha*p*4NAc-(1→ connected to two adaptor sugars and a primer →4)-α-D-Man*p*-(1→3)-α-D-Man*p*-(1→3)-β-D-Qui*p*NAc which links to the core, was generated by CarbBuilder v2.1.17 and visualized with PyMol 1.3. Methyl groups of *N*-acetyl groups in perosamine residues are highlighted in red color.

The substitution of the formamide by the acetamide group in perosamine resulted in a profound modification of the epitopic structure of the O-PS. For the *wbdR*Δ*wbkC* constructs, reactivity with monoclonal antibodies of C/Y and A>M (in *B. abortus*), or C/Y-A = M and M (in *B. melitensis*) specificity was severely hampered. This is consistent with the critical role of the formamide group in the interaction with the binding site of monoclonal antibody Yst9.1 (an anti-*Y. enterocolitica* O:9 antibody of C/Y reactivity) and with the observation that chemical removal of the formyl group followed by full *N*-acetylation reduces functional antibody affinities one thousand fold below those of the natural antigen (Caroff et al., 1984; Bundle, 1989; Oomen et al., 1991). For the *wbdR* constructs that conserved the formyl-transferase gene *wbkC*, the *N*-formyl plus *N*-acetyl-perosamine O-PS also lost the reactivity with the monoclonal antibodies tested. Since the biding sites of monoclonal antibodies of A, C/Y and M specificities have been estimated to accommodate five or less *N*-formyl-perosamine residues (Bundle, 1989; Oomen et al., 1991), the results strongly suggest that the acetamide groups in these O-PS are interspersed among the formamide ones thereby disrupting the interaction with the antibodies. Further NMR studies or the validation of the binding site of monoclonal antibodies by crystal structure analysis of complexes with oligosaccharides would be necessary to confirm this interpretation. The observations with monoclonal antibodies were complemented with the polyclonal sera because the anti-Ba-p*wbdR* serum and the absorptions conducted to obtain the Anti-Acetyl reagent demonstrated that the acetamide group was associated with the appearance of new epitope(s) absent from the wild-type strains. Taking into account that the acetamido group is larger than the formamido one, it remains to be analyzed in detail whether *N*-acetyl-perosamine O-PS induces antibodies that can still bind *N*-formyl-perosamine O-PS and their affinity. Topologically, previous molecular modeling and analysis of the antibody binding sites shows that the *N*-formyl-perosamine formamido group is exposed on *Brucella* wild-type O-PS (Bundle, 1989; Peters et al., 1990). Modeling using CarbBuilder (Kuttel et al., 2016) shows that in the *wbdR*Δ*wbkC* constructs the acetamide methyl group is exposed (**Figure 8**) thus replacing the formyl hydrogen atom of the 4-amido substituent of the wild-type O-PS, which accounts for the change in antibody reactivity to these O-PS.

One relevant question is to what extent if any the O-PS modifications described here alter properties that are associated with virulence, and this has been partially studied here. The O-PS is known to be critical in the marked resistance of S brucellae to the bactericidal action of complement in non-immune serum (Eisenschenk et al., 1995). This resistance results from a hindrance by *Brucella* O-PS of C1 access to the outer membrane proteins combined with the reduced activation of the complement cascade that depends on the structural peculiarities of its core and lipid A (Eisenschenk et al., 1999; Lapaque et al., 2005; Conde-Álvarez et al., 2012). The experiments presented in this work show that bacteria carrying N-acetyl-perosamine O-PS are more sensitive to normal serum than the wild-type strains. Studies with *E. coli* O111B4 and *Salmonella montevideo* have demonstrated that the resistance of these bacteria to normal serum correlates with the degree of O-PS polymerization and coverage of the cell surface by LPS (Goldman et al., 1984; Joiner et al., 1984; Grossman et al., 1987). Since the SDS-PAGE and Western-blot analyses show shorter O-PS for the *B. abortus wbdR* constructs than for the wild-type bacteria, this difference could account for the increased serum sensitivity of the former, in parallel to the observations in *E. coli* and *S. montevideo.* This hypothesis is compatible with the lack of an effect of *N*-acetyl-perosamine on the resistance to polymyxin B as the core and lipid A sections should not be affected by *wbdR*. Indeed, these are the sections involved in the resistance to bactericidal peptides in *B. abortus* and *B. melitensis* (Moreno et al., 1981; Conde-Álvarez et al., 2012; Fontana et al., 2016) and, in fact, a change in surface charge that could reveal an exposure of anionic groups was not detected. It is worth noting that serum sensitivity did not result in a significant decrease of the bacteria in their ability to multiply in the spleens of mice (and hence intracellularly in a variety of cells [Copin et al., 2012]) and to reach the chronic phase. Although this could be interpreted to mean that serum resistance is not relevant in S *Brucella* virulence, the result could also reflect the limitations of the mouse model and/or the route of infection in this model. Studies in sheep using *wbdR* and *wbdR*Δ*wbkC* modified *B. melitensis* Rev 1 vaccine are in progress to analyze these aspects and to study whether the structural modifications and epitopes detected under laboratory conditions are meaningful in these natural hosts. In this connection, it is also worth commenting on the potential practical application of the *wbdR* constructs, the main motivation of the present work.

The best available brucellosis vaccines (i.e., strains *B. melitensis* Rev 1 and *B. abortus* S19) carry O-PS and induce antibodies that interfere in the best diagnostic tests and thus in serological diagnosis (Ducrotoy et al., 2018). Although this problem can be significantly reduced by a judicious choice of the route and age of vaccination, the interference is troublesome. This problem is particularly important in Rev 1 because this vaccine is used not only against *B. melitensis* but also against *B. ovis*, an R species devoid of O-PS that infects sheep. Because of the serological interference, countries that have eradicated *B. melitensis* ban the use of Rev 1 and are thus without immunoprophylactic tools to combat *B. ovis*. Accordingly, it is generally acknowledged that a brucellosis vaccine that would not interfere with serodiagnosis, or a test that would discriminate antibodies to wild-type O-PS elicited by S vaccines and virulent S brucellae would represent a definite asset. Concerning the second possibility, suggestive work with synthetic oligosaccharides has been presented recently and, moreover, immunizations with the corresponding glyconjugates proposed for vaccination (Ganesh et al., 2014; Bundle and McGiven, 2017; Mandal et al., 2017). The results presented here suggest that *wbdR* could be used for differentiating the antibody response occurring during an infection by wild-type strains from that induced upon vaccination with *wbdR* modified strains. Since the method described here works in both *B. melitensis* and *B. abortus*, the background strain could be one of the existing S vaccines (i.e., *B. melitensis* Rev1 and *B. abortus* S19) but also a totally new vaccine. Concerning the ancillary serological test, there is also a variety of possibilities. Classical buffered *Brucella* antigen agglutination and complement fixation tests use whole bacteria and, as the O-PS is surface exposed, they could be implemented with cells from a *wbdR*-modified strain, more likely of the *wbdR*Δ*wbkC* type. Logistic difficulties posed by these tests can be circumvented using immunoenzymatic assays, and the LPS of the *wbdR* modified strains, or the hydrolytic polysaccharide if cross-reactivity at LPS core level poses a problem, are a clear alternative (Ducrotoy et al., 2016) Studies in natural hosts are in progress to investigate whether these tests combined with formamido-tagged vaccines are useful tools in the control of animal brucellosis.

## Author Contributions

RC-Á, MI, and IM conceived and coordinated the study. GW supervised the NMR studies. EM-G, YG-R, AZ-R, JS, MZ, MI, and RC-Á performed the experiments and genomic analyses. RC-Á, GW, and IM wrote the manuscript. All authors analyzed the results and approved the final version of the manuscript.

## Conflict of Interest Statement

RC-Á, AZ-R, MI, and IM are inventors of patent EP15201717.4 covering potential uses of *wbdR* constructs. The other authors declare that the research was conducted in the absence of any commercial or financial relationships that could be construed as a potential conflict of interest.

## Footnotes

1 http://www.ncbi.nlm.nih.gov/

## Abbreviations

CFU: colony forming units
Cm: chloramphenicol
HMBC: heteronuclear multiple-bond correlation
HSQC: heteronuclear single quantum coherence
Km: kanamycin
LPS: lipopolysaccharide
NMR: nuclear magnetic resonance
O-PS: O-specific polysaccharide
PAMP: pathogen associated molecular pattern
PS: O-PS-core oligosaccharide
R: rough
S: smooth
TOCSY: total correlation spectroscopy
TSA: tryptic soy agar.
