# Supplementary material for: Genomic Insertion of a Heterologous Acetyltransferase Generates a New Lipopolysaccharide Antigenic Structure in Brucella abortus and Brucella melitensis
Source: Front Microbiol. 2018 May 25;9:1092. doi: 10.3389/fmicb.2018.01092 (PMC5981137; doi:10.3389/fmicb.2018.01092)
Supplement: Supplementary file 1 [file Table_1.DOCX]

**Table S1.** Bacterial strains and plasmids

| **Strain/Plasmid** | **Relevant characteristics** | **Reference/ Source** |
| --- | --- | --- |
| ***Brucella abortus*** |  |  |
| Ba-parental | Nal^R^ spontaneous mutant of the 2308 strain received in 1983 from Dr. Lois M. Jones from the laboratory of Prof. David T. Berman (University of Wisconsin, Madison) as a lyophilized vial coming originally from the National Animal Disease Center at Ames, USA. It has been kept at the laboratory of the authors using the master seed strategy to minimize genetic drifts. It is virulent in mice and has been recently sequenced and found to differ from 2308 and 2308A (sequenced). Hence, the denomination "2308W" is adopted here. | Sangari and Agüero 1991  Suárez-Esquivel et al., 2016; |
| Ba-p*wbdR* | Ba-parental with a plasmid carrying the *wbdR* gene of *E. coli* O157:H7 | This work |
| Ba::Tn7*wbdR*Km^R^ | Ba-parental with miniTn7 transposon, carrying the *wbdR* gene of *E. coli* O157:H7 inserted in the chromosome | This work |
| Ba::Tn7*wbdR* | Ba-parental with miniTn7 transposon, carrying the *wbdR* gene of *E. coli* O157:H7 inserted in the chromosome. It carries an internal deletion in the kanamycin resistance gene Δ*km* _3-261_ | This work |
| Ba::Tn7*wbdR*Δ*wbkC* | Ba-parental with miniTn7 transposon, carrying the *wbdR* gene of *E. coli* O157:H7 inserted in the chromosome. It carries an internal deletion in the kanamycin resistance gene (Δ*km* _3-261_) and *wbkC* gene (Δ*wbkC* _30-211_) | This work |
| Ba*Δper* | Ba-parental *per*Δ _133-354_ rough-LPS mutant | This work (constructed as in González et al. 2008) |
| ***Brucella melitensis*** |  |  |
| Bme-parental | Nal^R^ spontaneous mutant of strain *B.melitensis 16M*; virulent; S-LPS | González et al. 2008 |
| Bme::Tn7*wbdR*Km^R^ | Bme-parental with miniTn7 transposon, carrying the *wbdR* gene of *E. coli* O157:H7 inserted in the chromosome | This work |
| Bme::Tn7*wbdR* | Bme-parental with miniTn7 transposon, carrying the *wbdR* gene of *E. coli* O157:H7 inserted in the chromosome. It carries an internal deletion in the kanamycin resistance gene Δ*km* _3-261_ | This work |
| Bme::Tn7*wbdR*Δ*wbkC* | Bme-parental with miniTn7 transposon, carrying the *wbdR* gene of *E. coli* O157:H7 inserted in the chromosome. It carries an internal deletion in the kanamycin resistance gene (Δ*km* _3-261_) and *wbkC* gene (Δ*wbkC* _30-211_) | This work |

| O157:H7 | *E. coli* strain carrying  *wbdR* gene | Spanish Type Culture Collection (CECT 4783) |
| --- | --- | --- |
| S17-1λpir | Mating strain with plasmid RP4 inserted into the chromosome | Simon, Priefer, and Puhler 1983 |
| Top10F´ | F´{lacIq, Tn10(TetR)} *mcrA* Δ(*mrr-hsdRMS-mcrBC*) Φ80*lacZ*ΔM15 Δ*lacX*74 *recA*1 *araD*139 Δ(*ara leu*) 7697 *galU galK rpsL* (StrR) endA1 nupG | Invitrogen |
| One shot OMNIMAX ™ | F´{proABlacIqlacZΔM15Tn10(TetR)Δ(*ccdAB*)mcrA Δ(*mrr hsdRMS-mcrBC*) Φ 80(*lacZ*)ΔM15 Δ(*lacZYA argF*)U169endA1recA1supE44thi1gyrA96relA1tonApa nD | Invitrogen |
| SM10λpir | *th*-1 *thr* *leu* *tonA* *lacY* *supE*, *recA*::RP4-2-Tc::Mu KmR (λpir) | Miller and Mekalanos 1988 |
| HB101 | F - *hsdS*20 *recA13* *ara*-14 *proA2* *lacY1* *galK2* *rpsL20* *xyl*-*5* *mtl*-1 *supE44* | Sambrook, Fritsch, and Maniatis 1989 |
| **Plasmids** |  |  |
| pCR2.1 | Cloning vector, Km^R^ | Invitrogen |
| pJQKm | Suicide vector, Km^R^, Sac^S^ | Scupham and Triplett 1997 |
| pDONR221 | Cloning vector containing attP recombination sites for BP reaction (Gateway system) | Invitrogen |
| pRH001 | Derivative of pMR10; Km^R^ Cm^R^ | Hallez et al. 2007 |
| pGEM®-T Easy | Cloning vector | Promega |
| pUC18R6KTminiTn7Tkm | pUC18R6KTminiTn7T with Km cassette | Llobet et al. 2009 |
| pRK2013 | Helper vector containing *tra* y *mob* genes . Col E1 replicon KmR | Figurski and Helinski 1979 |
| pTNS2 | Plasmid expressing *tnsABCD* from P*lac*. ApR | Choi et al. 2005 |
| pNPTS138-Cm | Cloning vector, Suicide plasmid, Cm^R^ | Addgene |
| pYRI-5 | 727-bp of *E. coli* O157:H7 chromosomal DNA containing the *wbdR* ORF and attB sites, cloned into pDONR221 | This work |
| pYRI-6 | Contains the fragment attL1-attL2 from pYRI-5 cloned into attR1-attR2 sites of pRH001 | This work |
| pYRI-21 | 727-bp of *E. coli* O157:H7 chromosomal DNA containing the *wbdR* ORF and 300-bp upstream, cloned into pGEM-T Easy | This work |
| pYRI-27 | 727-bp of *E. coli* O157:H7 chromosomal DNA containing the *wbdR* ORF and 300-bp upstream, cloned into pUC18R6KTminiTn7Tkm | This work |
| pCRΔKm | 780-bp of DNA of a deleted Kanamycin resistance gene generated by PCR and cloned into pCR2.1 | This work |
| pRCI-65 | *Eco*RI fragment of pCRΔKm cloned in the corresponding site of pNPTS138-Cm | This work |
| pYRI-30 | 827-bp of *B.abortus* chromosomal DNA containing the BAB1_0540 (*wbkC*) deletion allele, generated by PCR and cloned into pCR2.1 | This work |
| pYRI-31 | *Bam*HI*/Xba*I fragment from pYRI-30 cloned into the corresponding sited of pJQKm | This work |

Choi, Kyoung-Hee et al. 2005. “A Tn7-Based Broad-Range Bacterial Cloning and Expression System.” *Nature Methods* 2(6): 443–48. http://www.nature.com/doifinder/10.1038/nmeth765.

Figurski, David H, and Donald R Helinski. 1979. “Replication of an Origin-Containing Derivative of Plasmid RK2 Dependent on a Plasmid Function Provided in Trans (Plasmid Replication/replication Origin/trans-Complementation/broad Host Range/gene Cloning).” *Proc. Nati. Acad. Sc* 76(4): 1648–52.

González, David et al. 2008. “Brucellosis Vaccines: Assessment of Brucella Melitensis Lipopolysaccharide Rough Mutants Defective in Core and O-Polysaccharide Synthesis and Export.” *PLoS ONE* 3(7): e2760. http://dx.plos.org/10.1371/journal.pone.0002760.

Hallez, Régis, Jean Jacques Letesson, Jean Vandenhaute, and Xavier De Bolle. 2007. “Gateway-Based Destination Vectors for Functional Analyses of Bacterial ORFeomes: Application to the Min System in Brucella Abortus.” *Applied and Environmental Microbiology* 73(4): 1375–79.

Llobet, Enrique, Catalina March, Paloma Giménez, and José A Bengoechea. 2009. “Klebsiella Pneumoniae OmpA Confers Resistance to Antimicrobial Peptides.” *Antimicrobial Agents and Chemotherapy* 53(1): 298–302.

Miller, Virginia L, and John J Mekalanos. 1988. “A Novel Suicide Vector and Its Use in Construction of Insertion Mutations: Osmoregulation of Outer Membrane Proteins and Virulence Determinants in Vibrio Cholerae Requires toxR.” *Journal of Bacteriology* 170(6): 2575–83.

Sambrook, J., E.F. Fritsch, and T Maniatis. 1989. *Molecular Cloning. A Laboratory Manual, 2nd Ed*. 2 nd. ed. Cold Spring Harbor Laboratory Press. New York: Cold Spring Harbor,.

Sangari, F., and J. Agüero. 1991. “Mutagenesis of Brucella Abortus: Comparative Efficiency of Three Transposon Delivery Systems.” *Microb.Pathog* 11: 443–46.

Scupham, Alexandra J, and Eric W Triplett. 1997. “Isolation and Characterization of the UDP-Glucose 4-Epimerase-Encoding Gene, galE, from Brucella Abortus 2308.” *Gene* 202: 53–59.

Simon, R., U. Priefer, and A. Puhler. 1983. “A Broad Host Range Mobilization System for in Vivo Genetic Engineering: Transposon Mutagenesis in Gram Negative Bacteria.” *Nature Biotechnology* 1: 784–91.

Suárez-Esquivel, M., Ruiz-Villalobos, N., Castillo-Zeledón, A., Jiménez-Rojas, C., Roop II, R. M., Comerci, D. J., et al. (2016). *Brucella abortus* Strain 2308 Wisconsin Genome: Importance of the Definition of Reference Strains. *Front. Microbiol.* 7, 1–6. doi:10.3389/fmicb.2016.01557.
