# Supplementary material for: Genomic Insertion of a Heterologous Acetyltransferase Generates a New Lipopolysaccharide Antigenic Structure in Brucella abortus and Brucella melitensis
Source: Front Microbiol. 2018 May 25;9:1092. doi: 10.3389/fmicb.2018.01092 (PMC5981137; doi:10.3389/fmicb.2018.01092)
Supplement: Supplementary file 2 [file Table_2.DOCX]

**Table S2.** Primers

| **Primers** | **Sequence 5´-3´^1^** | **Comments** |
| --- | --- | --- |
| *wbdR* attB Fw | ggggacaagtttgtacaaaaaagcaggcttcATGAATTTGTATGGTATTTTTGGT | Used to amplify *wbdR* gene |
| *wbdR* attB Rv | ggggaccactttgtacaagaaagctgggtcTTAAATAGATGTTGGCGATCTT | Used to amplify *wbdR* gene |
| *wbdR* Fw | ttccccgggggagaagttcgccacagtaaatcgaa | Used to amplify *wbdR* gene |
| *wbdR* Rv | ttccccgggggattaaatagatgttggcgatctt | Used to amplify *wbdR* gene |
| GlmS_B | GTCCTTATGGGAACGGACGT | Used to verify the *wbdR* insertion |
| Ptn7-R | CACAGCATAACTGGACTGATT | Used to verify the *wbdR* insertion |
| Ptn7-L | ATTAGCTTACGACGCTACACCC | Used to verify the *wbdR* insertion |
| RecG | TATATTCTGGCGAGCGATCC | Used to verify the *wbdR* insertion |
| Tn7F | TGGCTAAAGCAAACTCTTCATTT | Used to verify the *wbdR* insertion |
| Tn7R | GCGGATTTGTCCTACTCAGG | Used to verify the *wbdR* insertion |
| *kmR*-F1 | AGGAAGCGGAACACGTAGAA | Used for overlapping PCR |
| *kmR*-R2 | AATCATGCGAAACGATCCTC | Used for overlapping PCR |
| *kmR*-F3 | **GAGGATCGTTTCGCATGATT**TTCTTCTGAGCGGGACTCTG | Used for overlapping PCR |
| *kmR*-R4 | TGGTCCATATGAATATCCTCCTTA | Used for overlapping PCR |
| *kmR*-R5 | CTTCCCGCTTCAGTGACAAC | Used to verify the *km* deletion |
| *wbkC*-F1 | AGGTGGCGACAAACGAATAA | Used for overlapping PCR |
| *wbkC-*R2 | GCCCATGCCAATCAAGGT | Used for overlapping PCR |
| *wbkC*-F3 | **ACCTTGATTGGCATGGGC** AGATGGTCGGAAGTCCAGATT | Used for overlapping PCR |
| *wbkC*-R4 | TCTGAACTCGGCTGGATGAC | Used for overlapping PCR |
| *wbkC*-R5 | GAGTTTGTTCCGCGATAAGC | Used to verify the *wbkC* deletion |

^1^In lowercase, *Sma*I Diana restriction

In underline, the start and the stop codon

In bold face, complementary region for overlapping PCR
