# Supplementary material for: Genomic Insertion of a Heterologous Acetyltransferase Generates a New Lipopolysaccharide Antigenic Structure in Brucella abortus and Brucella melitensis
Source: Front Microbiol. 2018 May 25;9:1092. doi: 10.3389/fmicb.2018.01092 (PMC5981137; doi:10.3389/fmicb.2018.01092)
Supplement: Supplementary file 3 [file Table_3.DOCX]

**Table S3.** Bacteria with O-antigen containing perosamine residues with *N*-acyl substitutions other than *N*-formyl.

|  | Perosamine characteristics | | | Additional sugars^1^ | References |
| --- | --- | --- | --- | --- | --- |
| Bacteria | Configuration | *N*-acyl Group | Linkage(s) [proportion] |  |  |
| *Vibrio cholerae* O76 | L | S-(+)-2-hydroxypropionyl | α (1,2) | - | (Kondo et al. 1996) |
| *V. cholerae* O1 Ogawa | D | 3-deoxy-L-glycero-tetronyl | α (1,2) | - | (Isshiki et al. 1995) |
| *V. cholerae* 1875 | D | 3-hydroxypropionyl | α (1,2) | - | (Kondo et al. 1993) |
| *V. cholerae* Hakata | D | acetyl | α (1,2) | - | (Isshiki et al. 1992) |
| *V. cholerae* O144 | L | *R*-(-)-2-hydroxypropionyl | α (1,2) | - | (Sano et al. 1996) |
| *Escherichia coli* O157:H7 | D | acetyl | α (1,2), α (1,3)^2^ | +++ | (Perry and Bundle 1990) |
| *E. hermannii*^3^ | D | acetyl | α (1,2), α (1,3) [3:2 o 2:3] | - | (Perry and Bundle 1990) |
| *Salmonella* group N | D | acetyl | α (1,2), α (1,3)^4^ | +++ | (Perry and Bundle 1990) |
| *Stenotrophomonas maltophila* | D | acetyl | α (1,2) α (1,3) [1:1] | + | (Perry and Bundle 1990) |
| *Citrobacter gillenii* O9a,9b | D | acetyl | α (1,2) | - | (Lipiński et al. 2002) |
| *Citrobacter youngae* O9 | D | acetyl | α (1,2), α (1,3), β (1,3) | - | (Ovchinnikova et al. 2004) |
| *Caulobacter crescentus*^5^ | Unknown | acetyl | Unknown | Possibly | (Awram and Smit 2001) |

^1^ +, presence; -, absence.

^2^ No perosamine - perosamine linkages.

^3^ Only in some serotypes [NRCC strain, but no ATCC strains (Perry and Bundle, 1990; Sano *et al.*, 1996)].

^4^ No perosamine - perosamine linkages.

^5^ Genetic evidence; no structural studies.

Awram, Peter, and John Smit. 2001. “Identification of Lipopolysaccharide O Antigen Synthesis Genes Required for Attachment of the S-Layer of Caulobacter Crescentus.” *Microbiology* 147: 1451–60.

Isshiki, Yasunori, Yuji Haishima, Seiichi Kondo, and Kazuhito Hisatsune. 1992. “Serological Cross-Reaction between Intact and Chemically Modified Lipopolysaccharides of O1 Vibrio Cholerae Inaba and Non-O1 V. Cholerae Bio-Serogroup Hakata.” *Microbiology and Immunology* 36(11): 1201–5.

Isshiki, Yasunori, Yuji Haishima, Seiichi Kondo, and Kazuhito Hisatsune. 1995. “Immunochemistry of Group A and Inaba C Antigen Factors Constituting the 0 Antigen of O1 Vibrio Cholerae.” *European Journal of Biochemistry* 229: 583–88.

Kondo, Seiichi et al. 1993. “N-3-Hydroxypropionyl-α-D-Perosamine Homopolymer Constituting the O-Chain of Lipopolysaccharides from Vibrio Bioserogroup 1875 Possessing Antigenic Factor(s) in Common with O1 Vibrio Cholerae.” *Biochem. J* 292: 531–35.

Kondo, Seiichi, Yasuhiro Sano, Yasunori Lsshiki2, and Kazuhito Hisatsunel. 1996. “The 0 Polysaccharide Chain of the Lipopolysaccharide from Vibrio Cholerae O76 Is a Homopolymer of N-[(S)-(+)-2- Hydroxypropionyl]-a-L-Perosamine.” *Microbiology* 142: 2879–85.

Lipiński, Tomasz et al. 2002. “Structures of Two O-Chain Polysaccharides of Citrobacter Gillenii O9a,9b Lipopolysaccharide.” *European Journal of Biochemistry* 269(1): 93–99. http://doi.wiley.com/10.1046/j.0014-2956.2001.02638.x (October 5, 2016).

Ovchinnikova, Olga G et al. 2004. “Structures of Two O-Polysaccharides of the Lipopolysaccharide of Citrobacter Youngae PCM 1538 (Serogroup O9).” *Carbohydrate Research* 339: 881–84.

Perry, Malcolm B, and David R Bundle. 1990. “Antigenic Relationships of the Lipopolysaccharides of Escherichia Hermannii Strains with Those of Escherichia Coli O157:H7, Brucella Melitensis, and Brucella Abortus.” *Infection and Immunity*: 1391–95.

Sano, Yasuhiro et al. 1996. “An N-[(R )-(-)-2-Hydroxypropionyl]-α-L-Perosamine Homopolymer Constitutes the O Polysaccharide Chain of the Lipopolysaccharide from Vibrio Cholerae O144 Which Has Antigenic Factor(s) in Common with V. Cholerae O76.” *Microbiology and Immunology* 40(10): 735–41. http://doi.wiley.com/10.1111/j.1348-0421.1996.tb01134.x (October 5, 2016).
