# Supplementary material for: Genomic Insertion of a Heterologous Acetyltransferase Generates a New Lipopolysaccharide Antigenic Structure in Brucella abortus and Brucella melitensis
Source: Front Microbiol. 2018 May 25;9:1092. doi: 10.3389/fmicb.2018.01092 (PMC5981137; doi:10.3389/fmicb.2018.01092)
Supplement: Supplementary file 4 [file Table_4.DOCX]

**Table S4**. ^1^H and ^13^C NMR chemical shifts (ppm) in D_2_O at 25 ᵒC for Ba-parental PS.

| →2)-α-d-Rha*p*4NFo-(1→ |  | 1 | 2 | 3 | 4 | 5 | 6 | Formyl |
| --- | --- | --- | --- | --- | --- | --- | --- | --- |
|  | s-*cis* (~80%) | 5.186 | 4.168 | 4.103 | 3.994 | 3.881 | 1.216 | 8.216 |
|  |  | 101.33 | 77.79 | 68.38 | 52.65 | 69.06 | 17.66 | 165.72^a^ |
|  | s-*trans* (~20%) | 5.186 | 4.168 | 4.069 | 3.438 | 3.927 | 1.269 | 8.048 |
|  |  | 101.33 | 77.79 | 68.26 | 57.69 | 68.48 | 17.48 | 168.65 |

^a^ A minor peak at δ_C_ 165.27 attributed to a 3-linked residue was also present.
