# Supplementary material for: Genomic Insertion of a Heterologous Acetyltransferase Generates a New Lipopolysaccharide Antigenic Structure in Brucella abortus and Brucella melitensis
Source: Front Microbiol. 2018 May 25;9:1092. doi: 10.3389/fmicb.2018.01092 (PMC5981137; doi:10.3389/fmicb.2018.01092)
Supplement: Supplementary file 5 [file Presentation_1.PDF]

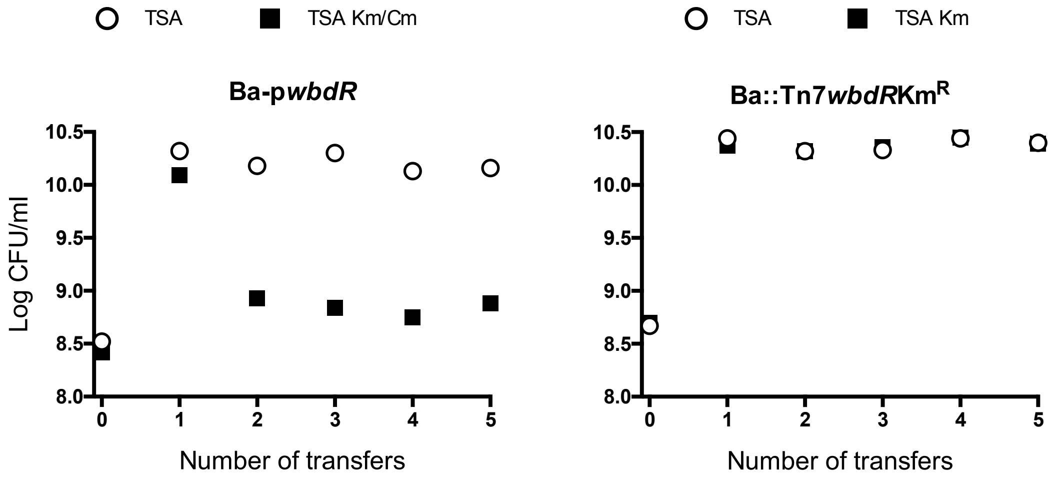

Figure S1. Insertion of *wbdR* into chromosome II generates a stable Ba::Tn7*wbdR* construct. Ba-p*wbdR* (left panel) and Ba::*Tn7wbdR*Km^{R} (right panel) stability was studied by serial passage in broth without antibiotics, and CFU/ml determined on TSA (controls), TSA Km/Cm (p*wbdR* plasmid markers) or TSA Km (Tn7*wbdR* marker).
