# Supplementary material for: Genomic Insertion of a Heterologous Acetyltransferase Generates a New Lipopolysaccharide Antigenic Structure in Brucella abortus and Brucella melitensis
Source: Front Microbiol. 2018 May 25;9:1092. doi: 10.3389/fmicb.2018.01092 (PMC5981137; doi:10.3389/fmicb.2018.01092)
Supplement: Supplementary file 6 [file Presentation_2.PDF]

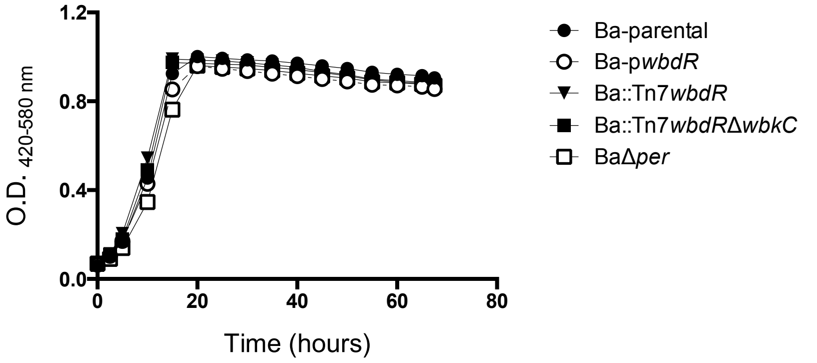

Figure S2. The *wbdR* constructs do not show growth defects *in vitro*. Ba-parental, Ba-p*wbdR*, Ba::Tn7*wbdR*, Ba::Tn7*wbdR*Δ*wbkC* and the rough mutant BaΔ*per* as a control were grown in TSB. Each point represents the mean of triplicates samples (error bars are within the size of the symbols). Data are representative of three independent experiments.
