# Supplementary material for: Genomic Insertion of a Heterologous Acetyltransferase Generates a New Lipopolysaccharide Antigenic Structure in Brucella abortus and Brucella melitensis
Source: Front Microbiol. 2018 May 25;9:1092. doi: 10.3389/fmicb.2018.01092 (PMC5981137; doi:10.3389/fmicb.2018.01092)
Supplement: Supplementary file 7 [file Presentation_3.PDF]

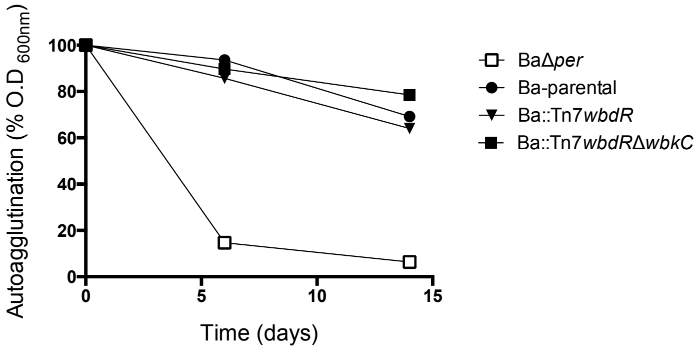

Figure S3. *wbdR* constructs do not autoagglutinate (Ba-parental and the R-mutant BaΔ*per* are the negative and positive autoagglutination controls, respectively)
