# Supplementary figures and images for: Genomic Insertion of a Heterologous Acetyltransferase Generates a New Lipopolysaccharide Antigenic Structure in Brucella abortus and Brucella melitensis
Source: Front Microbiol. 2018 May 25;9:1092. doi: 10.3389/fmicb.2018.01092 (PMC5981137; doi:10.3389/fmicb.2018.01092)

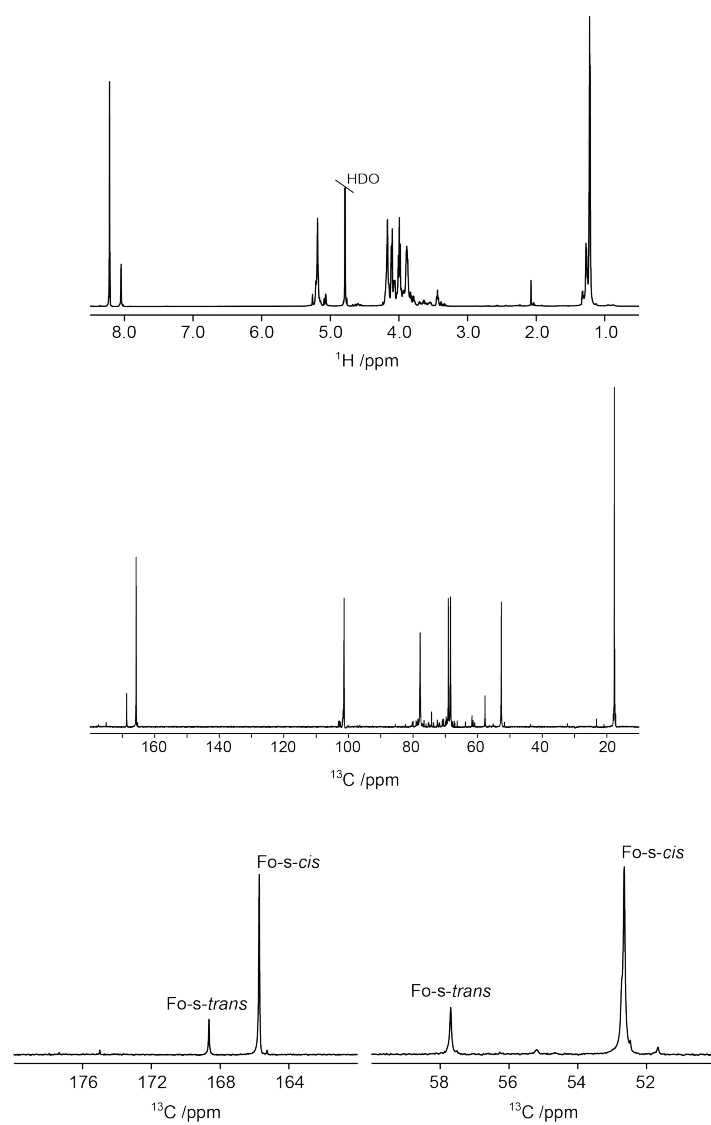

**Figure S4.**  $^1\text{H}$  and  $^{13}\text{C}$  NMR spectra of Ba-parental PS.

Supplement: Supplementary file 8 [file Presentation_4.PDF]
