# Supplementary material for: Genomic Insertion of a Heterologous Acetyltransferase Generates a New Lipopolysaccharide Antigenic Structure in Brucella abortus and Brucella melitensis
Source: Front Microbiol. 2018 May 25;9:1092. doi: 10.3389/fmicb.2018.01092 (PMC5981137; doi:10.3389/fmicb.2018.01092)
Supplement: Supplementary file 9 [file Presentation_5.PDF]

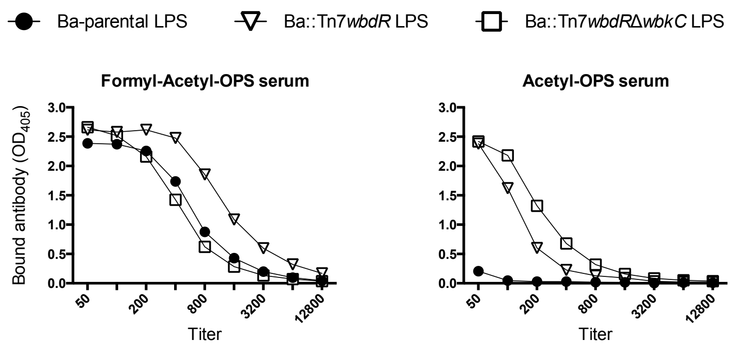

Figure S5. Absorption with cells of the R mutant BaΔ*per* does not affect the reactivity of the antisera in S-LPS iELISAs
