# Supplementary material for: Genomic Insertion of a Heterologous Acetyltransferase Generates a New Lipopolysaccharide Antigenic Structure in Brucella abortus and Brucella melitensis
Source: Front Microbiol. 2018 May 25;9:1092. doi: 10.3389/fmicb.2018.01092 (PMC5981137; doi:10.3389/fmicb.2018.01092)
Supplement: Supplementary file 10 [file Presentation_6.PDF]

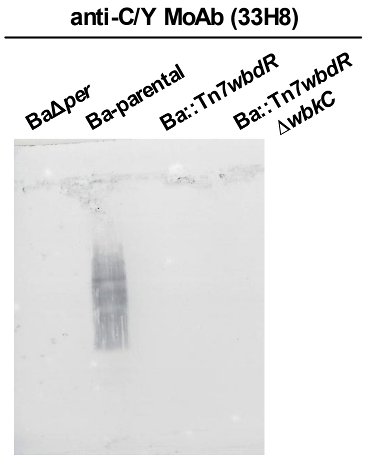

Figure S6. *N*-acetyl-perosamine alters the C/Y epitope characteristic of the *Brucella* O-PS. Western blot of LPS extracts with an anti-C/Y monoclonal antibody.
