# Supplementary material for: Genomic Insertion of a Heterologous Acetyltransferase Generates a New Lipopolysaccharide Antigenic Structure in Brucella abortus and Brucella melitensis
Source: Front Microbiol. 2018 May 25;9:1092. doi: 10.3389/fmicb.2018.01092 (PMC5981137; doi:10.3389/fmicb.2018.01092)
Supplement: Supplementary file 11 [file Presentation_7.PDF]

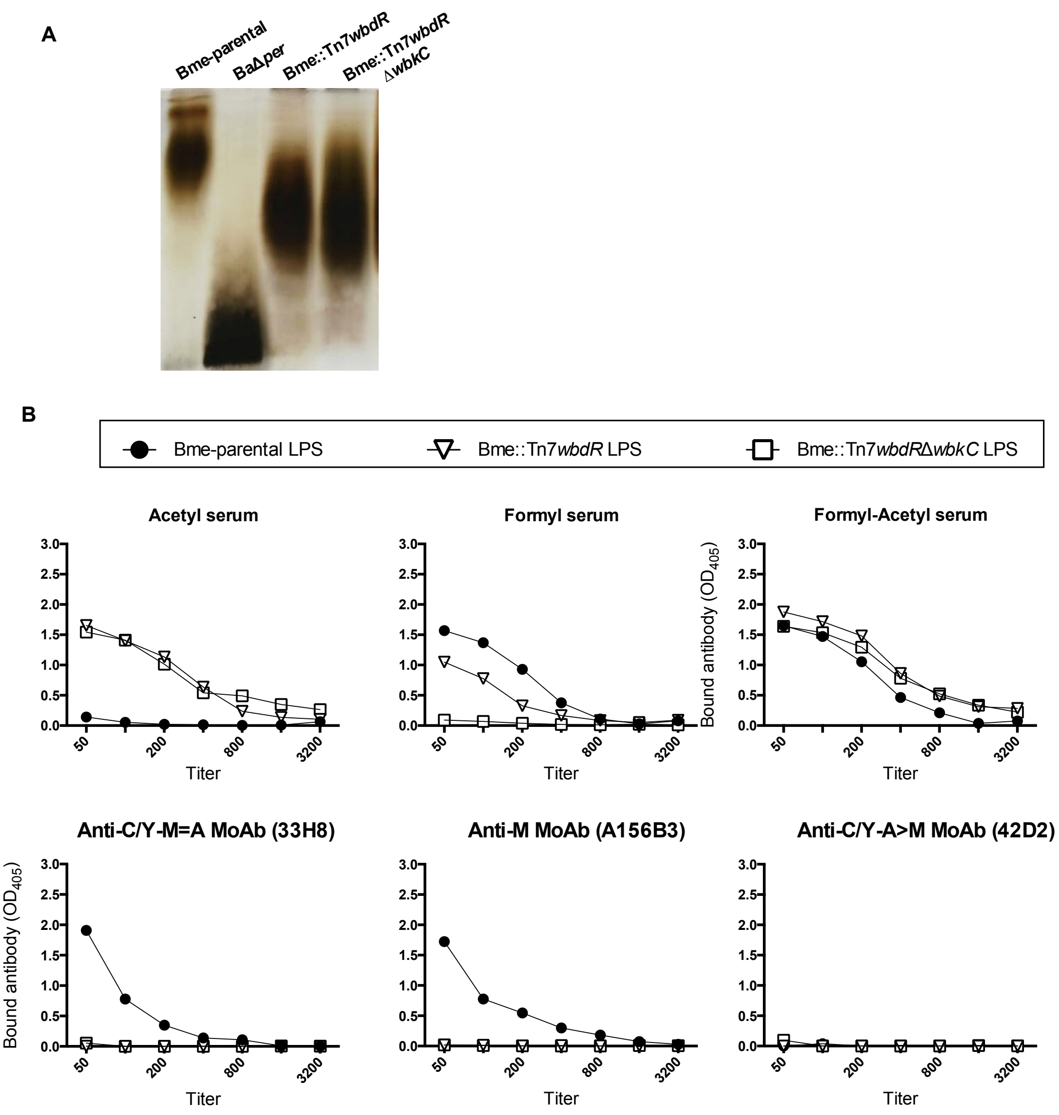

Figure S7. The *wbdR*-encoded acetyltransferase is also active in *B. melitensis* A) *B. melitensis wbdR* constructs display shorter O-PS**.** SDS-PAGE of LPS extracts followed by silver staining. B) *N*-acetyl-perosamine generates new epitopes in the S-LPS alters the reactivity of S-LPS with monoclonal antibodies of C/Y-A=M, C/Y-A>M and M specificities. ELISA with the *B. melitensis wbdR*-constructs and the indicated sera or monoclonal antibodies
